# International trauma care: initial European approaches during the COVID 19 pandemic

**DOI:** 10.1097/OI9.0000000000000112

**Published:** 2021-03-15

**Authors:** Tim J.S. Chesser, Robert Handley, Johannes Kloos, Gerrit De Wachter, Guy Putzeys, Jesús Gómez-Vallejo, Coral Sánchez-Pérez, Francisco Chana-Rodríguez, Filippo Raggini, Carlotta Pari, Stefania Paderni, Achille Contini, Alberto Belluati MD, Ioannis Daskalakis, Ioannis Sperelakis, Athanasios Kostakos, Theodoros H. Tosounidis, Sascha Halvachizadeh MD, Hans-Christoph Pape MD, Bertil Bouillon, Berend-Jan de Bruin, Keesjan J. Ponsen

**Affiliations:** a North Bristol NHS Trust, Southmead Hospital, Bristol; b John Radcliffe Hospital, Oxford, UK; c Orthopaedic and Traumatology Surgeon, Jessa Hospital, Hasselt; d Orthopaedic and Traumatology Surgeon, Groeninge Hospital Kortrijk, Belgium; e Orthopaedic Department, General University Hospital Lozano Blesa, Zaragoza; f Orthopaedic Department, General University Hospital Gregorio Marañón, Madrid, Spain; g Orthopaedic and Trauma Department, University Vita-Salute San Raffaele, Milan; h Orthopaedic and Trauma Department, Hospital Santa Maria delle Croci, Ravenna; i Orthopardic and Trauma Department, Ospedale del Mare, Naples; j Orthopaedic and Trauma Department, Hospital Santa Maria delle Croci, Ravenna, Italy; k Orthopaedic Department; l Orthopaedic Department, University Hospital of Heraklion, Crete; m 1st Orthopaedic Department, KAT Hospital, Athens; n School of Medicine, University of Crete, Crete, Greece; o Department of Trauma, University Hospital Zurich; p Harald-Tscherne Laboratory for Orthopedic and Trauma Research, University of Zurich, Zurich, Switzerland; q Department of Trauma and Orthopaedic Surgery, University of Witten/Herdecke, Cologne Merheim Medical Center, Cologne, Germany; r Department of Surgery; s Department of Surgery, TraumaUnit, Noordwest ZH Groep, Alkmaar, the Netherlands

**Keywords:** trauma, COVID19, coronavirus

## Abstract

The world was not prepared for the global of pandemic in early 2020 with the arrival of COVID 19. Europe has some of the most developed health care systems in the world and this article explains the initial response to the pandemic from an orthopaedic and trauma viewpoint from 8 nations. Italy reported the first cluster in February, which then rapidly spread around the continent, requiring a rapid reorganization of services. The reports highlight how elective surgery was universally stopped, surgical services were reconfigured, and new practices, such as the widespread use of telemedicine, may well become permanent. It also emphasizes how the pandemic has re-educated us on the importance of a consistent and central approach to deal with a global health crisis, and how medical services need to remain flexible and responsive to new ways of working.

## 1 Introduction

Europe developed its first cases of COVID-19 in late January, 2020, exacerbated by clusters in Northern Italy and Madrid, which rapidly spread across the continent. While the European borders are freely open, each country is responsible for its own health care system. Reports from 8 countries are combined (United Kingdom, Belgium, Spain, Italy, Greece, Switzerland, Germany, and Netherlands). Each health system had to rapidly adapt and learn from each other, and due to the continuing evolving situation, this report is a position of each country's orthopaedic and trauma's services response at the end of June, 2020. At the time of writing, each country was dealing with local outbreaks at the same time while reintroducing normal services.

## 2 United Kingdom

Whilst the countries comprising the United Kingdom reacted in a similar manner to COVID-19 this information applies primarily to England. Through February and March 2020, the public experienced a period of progression through awareness, interest, anxiety, and fear with a consequent motivation of preparation. A perceived advantage of a unified system of health care is that it allows for a coordinated consistent response to problems. On March 3, the headlines included: “*NHS bosses have declared coronavirus as a ‘level 4 incident’ — a move which allows NHS England to take command of all NHS resources across England*.” There was hesitation in the general political national action, and it was on March 23 that the lockdown in the UK began. On that day, there were just under 1000 new positive tests and 74 COVID deaths reported. However, health planning as a response to an impending surge in Coronavirus infections and admissions had begun before this time. Naturally, the major focus was on dealing with the direct respiratory problems of COVID-19, but there was a realization that all heath care services, including the trauma-related services, needed to be ready.

### 2.1 Organizational actions made to the trauma services

The British Orthopaedic Association posted guidance under the title “Orthopaedic Trauma and COVID-19” on its website on March 16, 2020 that appeared on NHS England's website a few days later.^[1]^ This document was subsequently modified to include references to nontrauma work and remained as NHS England's guidance. The aim of the initial guidance was to encourage that essential trauma work carried on, but to do so in a way that put a minimum demand on resources and took into account the safety of patients and staff. Physicians needed to continue to be advocates for their patients. It was predicted that the areas of the NHS that would need the greatest protection from unnecessary work were the emergency departments and those involved in respiratory support, particularly anesthetists. Consequently, recommendations were made to divert musculoskeletal injury away from emergency departments directly to Trauma and Orthopaedic services. Similarly, to reduce the load on operating theaters and, with the likelihood of anesthetist being redeployed to the airway management of COVID patients and intensive care, greater consideration was to be given to nonoperative management of fractures.

There are no reliable general activity figures for orthopaedic trauma. Even comparisons within a single geographic region are difficult as often trauma patients followed different pathways and were treated in different facilities as part of the reorganization. Two surveys were carried out by the British Orthopaedic Association utilizing the network of British Orthopaedic Clinical Directors Society (BODS); the first on the weekend of March 28 and the second on the weekend of May 15. In the first survey 54 hospital trusts responded. All reported that performing elective surgical procedures had ceased, with only 2 having had some elective clinical consultations. The prepandemic norm in England for the initial receipt of a patient with limb injuries was that they were seen by emergency department staff and then referred as appropriate to trauma and orthopaedics. By the emergence of the pandemic, 30% of patients with limb injuries bypassed the emergency department completely. In well over half of hospitals, the initial first contact assessment was carried out by trauma and orthopaedic consultants, which represented a complete change of practice from a few weeks before.

In a follow-up study being organized by NHS England on the potential beneficial changes that may be continued post COVID, early senior physician input into patient management had the most support. In a recent webinar organized by the British Orthopaedic Foot & Ankle Society, 1 presentation on the potential for post-COVID-19 increases in malunions and mistreated fractures was countered with a suggestion that the more frequent the involvement of senior surgeons in early injury management, the fewer the problems that may arise. Another notable finding from this early survey was the interest in and anxiety relating to personal protection equipment (PPE) with a clear desire for greater central guidance and clarity as to what precautions should be taken. Additionally, there were a multitude of questions relating to PPE.

By the time of the second survey,^[2]^ the general environment in most trusts had changed. There was a significant decline in the number of ventilated COVID-19 patients, yet, there was no resumption of normal workflow. PPE remained a significant issue; 10% of trusts reported that they did not have sufficient PPE for theater use. However, what had become particularly evident were the consequences that the required PPE and theater precautions took on the time to complete surgical procedures. Seventy-six trusts responded to the second survey. One question asked surgeons to estimate the increase in resources required for surgical procedures. Beginning with a baseline of what previously could have been completed in 10 surgical lists, surgeons were asked to estimate what resources were required under their current conditions. The responses demonstrated that the work of 10 surgical lists prepandemic, required 16 to be available postpandemic. In an environment where some staff members were absent, sick, or quarantining, these obstacles were compounded.

In an attempt to guide units in prioritizing their surgical work, a cross specialty prioritization document was drawn up by the Royal Colleges of Surgeons.^[3]^ This document has helped provide some structure to the progressive expansion of surgical activity as resources increasingly become available. However, as can be imagined, cross-specialty agreement has not been easy to achieve.

Some definitions used were confusing. The word “suspected” seemed to have 2 meanings in relation to COVID-19. For the medical patient attending the Emergency Department, it was someone who exhibited symptoms compatible with COVID-19; whereas for a surgical patient, it appeared to be anyone who was not proven to be negative. The definition of an aerosol-generating procedure in relation to COVID-19 was particularly problematic with Public Health England not introducing a distinction to include the source of the aerosol when using power tools until June 18, 2020.^[4]^ The aerosol-generating procedure definition changed from bone drilling or cutting to that only when working on the respiratory tract or para-nasal sinus. This, naturally, has had very significant implications for trauma and orthopaedic surgery, reducing the requirements for PPE and allowing improved theater efficiency.

In summary, in the early stages of the pandemic, there was a ready acceptance by both the NHS and practitioners that control would be centralized. As time has passed, there has been less willingness to produce generalized guidance, which has led to hesitation, uncertainty, and significant variations in practice around the country. There is, however, a great desire to make the most of any advances in practice and, in particular, the opportunities for change that the crisis has brought. As noted above in the initial surveys, there has been support for early definitive decision-making for patients with early input of senior staff. The other message that has been emphasized by many is that whilst preparation for the “worst” is appropriate and necessary, this is not synonymous with ceasing the normal care of patients. This issue must continue to be appropriately prioritized and continued where possible.

## 3 Belgium

Belgium is a small but centrally located country with a high population density of 373 inhabitants per km^2^.^[5]^^,^^[6]^ On July 1, 61,598 cases of COVID-19 (5.39 cases per 1000 inhabitants) were reported since the beginning of the pandemic, whereof 28.8% were hospitalized. Twelve percent of the hospitalized patients were admitted to the intensive care unit (ICU).^[7]^

With 9761 deaths through July 1, Belgium has one of the highest reported mortality rates (case-fatality ratio of 15.85%).^[8]^ Thirty-nine percent of the reported deaths were not confirmed by reverse transcription polymerase chain reaction and/or chest computed tomography (CT) due to the initially restricted testing policy, but remains registered as probable COVID-19-related deaths following WHO guidelines.^[7]^^,^^[9]^

The first lethal case in Belgium was reported on March 10 and, about 1 month later, the highest 1-day mortality due to COVID-19 was registered (343 deaths on April 12).^[7]^ The early days of the pandemic were marked by a narrow case definition (fever as a mandatory symptom) leading to restrictive testing, the scarce availability of PPE, and a single reference laboratory for the whole country.

Since then, numbers have been decreasing in a slow but steady way, with an average drop in incidence and mortality of 10% per week. On July 1, 203 patients were hospitalized in Belgium with 37 cases in the ICU, and an average of 85 new confirmed cases were registered per day during the week before July 1.^[5]^

This evolution is the result of a progressive lockdown^[10]^ imposed by the National Security Council starting on March 14, including the closure of schools and restaurants. From March 18, nonfood businesses closed as well and nonessential trips (except for food purchases and work) were prohibited. Country borders were closed for nonessential trips on March 20.^[11]^

In the health care sector, all nonessential surgeries and outpatient clinics were cancelled beginning on March 14 to save resources and staff for the emergency room (ER) and intensive care unit (ICU). A strict visitor restriction was applied to all hospitals and health care facilities.^[12]^ Recovery areas of the operating rooms (OR) were transformed into ICUs and operating room staff were retrained to run them, leading to a decrease in surgery-related resources such as scrub nurses, respirators, anesthetics, surgical masks, and gowns.

As an example, Jessa hospital is an accredited regional institution with 981 beds, 45,391 emergency admissions, and 41,183 surgical interventions in 2018, accounting for the 5th biggest nonuniversity hospital in the country.^[13]^ It is situated in the capital city of Limburg, the hardest hit province of Belgium (7.54 cases per 1000 inhabitants).^[7]^ During the peak period in the second week of April, 134 patients were hospitalized in this center and 31 out of 40 patients in the ICU needed invasive ventilation. Surgical capacity dropped from 21 to 4 functional ORs.

In this article, to demonstrate specific management protocols and challenges, the management of surgical trauma cases in this center during the early phase of the lockdown period, from March 14 to May 4, is described.^[14]^

### 3.1 Organizational actions made to the trauma services

Alarmed by the dramatic evolution in Northern Italy, a pathway to separate potential COVID-19 patients from those without suspicious symptoms was rapidly developed using container shelter-in-places and a designated radiology area for X-ray and CT scan.

On March 5, the case definition had been broadened and the laboratory testing capacities were increased by the federal authorities, and screening became mandatory for every patient being hospitalized in our center on April 10. Chest CT was initially added to increase sensitivity^[15]^ until the prevalence of positive radiologic findings dropped under 2%.^[16]^

Trauma patients admitted to the ER showing symptoms compatible with the early case definition of COVID-19 (fever and cough or dyspnea) were separated as early as possible and isolated individually in designated ER rooms. Health care providers (HCPs) working in these areas were equipped with PPE composed of filtering face piece (FFP)2/N95 masks, level 1 gowns, gloves, and face shields. FFP3/N99 masks were only used during swab testing, intubation (in ER, OR, and ICU), and level 3 or 4 gowns were only used in situations with high risk of contact with patient's body fluids (wound care, etc) following guidelines.^[17]^ Once PPE availability increased, every HCP at the ER was equipped with FFP2/N95 masks.

### 3.2 Hospitalization units

Tested patients were kept isolated and transferred from the ER to a COVID-19-specific transit hospitalization unit awaiting their results before being redirected to their definitive hospitalization unit. Patients not fulfilling all criteria for testing were immediately hospitalized at the trauma ward without transit hospitalization. Once the availability of surgical masks was increased, HCPs at the non-COVID-19 wards as well as negatively tested patients were asked to wear one if social distancing of 1.5 m could not be respected. Gloves were not advised, except for actions with potential contact with patient's body fluids.

Life- and limb-threatening emergencies as defined by the AAOS^[18]^ were transferred to a COVID-19-designated OR without further delay, where patient recovery after surgery was supervised until transfer to the transit hospitalization unit was possible, awaiting test results.

SARS-CoV-2-positive patients were hospitalized in single rooms (with an air lock if available) on dedicated wards, staffed with HCP wearing the same PPE as their colleagues in the ER. For all patients, the hospital stay was kept as short as possible. The use of calls/video calls was encouraged to keep contact with family and friends.

### 3.3 Operating room organization

Trauma patients with urgent surgical indications and a negative test result were cleared for surgery for the next 48 hours. If surgery needed to be postponed for more than 48 hours for any reason, a new reverse transcription polymerase chain reaction test had to be performed. The OR designated for surgery on COVID-19 infected or suspected patients, including life- and limb-threatening emergencies, was situated on another floor and equipped with an air lock and negative air pressure. Operating room staff wore FFP2/N95 masks, level 3 or 4 gowns (depending on the type of surgery), 2 pairs of surgical gloves, and face shields. A second circulating nurse was deployed to pass needed supplies to the air lock. Interventions on patients with negative test results were performed in 1 of the 3 remaining clean ORs, with HCPs wearing surgical masks, level 3 or 4 gowns, surgical gloves, and goggles.

### 3.4 Ambulatory follow-up

Urgent consultations for nonsurgical trauma patients (wound and plaster clinics) and essential postoperative consultations continued with reduced capacity to diminish waiting room traffic and provide time to disinfect used instruments, surfaces, and examination tables. Both patients and HCPs wore surgical masks, but the systematic use of gloves was discouraged.

COVID-19 patients were scheduled on specific dates in designated facilities, allowing coordination of logistics and radiology department to isolate the patient at every single step throughout the hospital. They wore surgical masks, whereas HCPs wore FFP2/N95 masks, level 1 gowns, gloves, and face shields. Isolation measures were taken during a period of 5 weeks after the first positive test result. Remote consultations were not used for the early follow-up of trauma patients.

### 3.5 Other important/unique experiences and lessons learned

With the restart of nonessential consultations and elective surgery from May 4,^[14]^ the page was turned and a new way of working with COVID-19 was to be defined. Although it is impossible to quantify the effect of these measures, so far none of the orthopaedic surgeons working during the crisis have contracted the virus. This provides HCPs with the confidence that these measures have been highly effective, but certainly further research is needed to establish the efficacy of each of these measures.

## 4 Spain

Since the pandemic was declared on March 11, 2020, the environment in Spain underwent totally unimaginable changes from just a few weeks prior.^[19]^ The pandemic began with a few cases scattered in larger cities and expanded throughout the nation asymmetrically, with Madrid becoming the epicenter of the pandemic in mid-March. Spain has been one of the countries with the highest mortality rates, with approximately 1000 deaths per day in the first week of April. Twenty percent of health workers tested positive, posing problems for the health care force and return to hospital normal activities. The data published on July 6, 2020 by the Spanish Ministry of Health showed a total number of 251,789 confirmed infected cases, 125,572 required hospital admission, 11,706 required admissions to an intensive care unit (there are a total of 4404 beds across Spain under normal circumstances), and a total number of 28,388 deceased patients.^[20]^

### 4.1 Organizational actions made to the trauma services

During the peak of the COVID-19 pandemic, the orthopaedic and traumatology surgeons could mainly impact 3 areas: avoiding the unnecessary use of overburdened facilities; preventing the exhaustion of resources; and controlling and protecting patients and health care workers.

The Spanish Society of Trauma and Orthopaedic Surgery (SECOT) published a guide with recommendations to facilitate these goals.^[21]^ Urgent surgical cases continued, while all nonurgent surgeries were suspended at most hospitals, and same-day surgery was encouraged, minimizing the unnecessary use of hospital beds.^[22]^ These steps preserved critical resources, including hospital beds, supplies, and personnel. As nonurgent surgery was reduced, these surgical facilities, equipment, and staff were reallocated to address COVID-19 patients.^[23]^

### 4.2 Emergency services

When patients entered an emergency room, they were screened for COVID-19 according to the standards of the Spanish Ministry of Healthcare. The patients with negative tests were taken to a segregated area for nonsuspicious cases of COVID-19, where standard care is provided in a COVID-19-free area, including that confirmed radiologically, with universal PPE usage.

If the patient's presenting condition required hospital admission from the emergency department and/or urgent surgery, all patients were screened with a PCR test and a chest x-ray before their admission. In patients classified as probable or positive COVID-19 cases, they were transferred to an isolation area and followed primarily by a COVID-19 specialist, with the help of a trauma team for stabilization; again with complete PPE use. If a patient presented after polytrauma and their COVID-19 status was unknown, the patient was treated as if they were positive, performing all the tasks with PPE and in a specialized room. Chest imaging with a CT scan additionally was helpful for the screening of the disease.^[24]^

### 4.3 Hospitalization

In those hospitals where trauma patients coexisted with COVID-19 patients, COVID-19-free areas, where patients and health workers were unlikely to develop the disease, were created, recognizing that negative PCR tests did not guarantee the absence of disease (false-negative PCR tests have been reported to be as high as 30%).^[25]^ If the center was specialized for COVID-19 care or had most of its facilities available for the treatment of this disease, urgent surgery was discouraged and referral to another facility was recommended. For the classification of areas, a triple nomenclature classification was recommended by SECOT, as the common classification of just 2 areas (COVID-19 and non-COVID-19) does not take into account the presence of external personnel in the health care center. This classification was as follows: Green area: Patient, staff, and material spaces shared by Non-COVID-19 cases according to the Spanish Ministry of Health; Yellow area: Outpatient, external staff, or visitors transit spaces, such as entrance halls or corridors, that cannot be guaranteed to be used only by Non-COVID-19 individuals; and Red area: COVID-19 positive, probable or possible patient spaces; these areas were isolated and never in contact with green areas.

### 4.4 Operating rooms

As a general rule, each emergency was treated as it would normally be treated pre-COVID-19, but with the spaces prepared for each patient according to their COVID-19 classification. Delay in the result of a test was not an excuse to delay the treatment of an urgent condition. COVID-19-negative patients found in the “green circuit” were treated with the standard rules for each operating room. With regards to face protection, if the surgical procedure produced aerosols, N95/ffp2-3 mask protection was used together with surgical or bidirectional masks.

Operating rooms, corridors, and other rooms in the operating room area were green areas. Surgery was carried out according to the general rules of each hospital. PPE was recommended for standard cases with aerosol production during the surgical process with utilization of a double mask to avoid contamination of the surgical field. Unless there were contraindications, the typical surgical techniques were not changed. Long-lasting dressings and wound closure techniques were utilized when possible.

### 4.5 Outpatient and posthospital care

Studies in other specialties have demonstrated the successful use of telemedicine during the COVID-19 pandemic.^[26]^ SECOT has provided recommendations for the establishment of a telemedicine program for those services where this resource had not previously been established. The benefits of telemedical consultation for trauma and orthopaedic surgery have already been demonstrated with excellent patient perceptions.^[27]^^,^^[28]^ The General Data Protection Regulations that apply in the European Union include a clause accounting for work done in the public's interest.^[29]^

### 4.6 Other important/unique experiences and lessons learned

Orthopaedic care in environments where COVID-19 care was provided evolved during the epidemic peak for COVID-19 in Spain. Standard, pre-COVID management protocols needed to change, making them more complex, with a reduction in elective care and focus on the treatment of emergent and urgent musculoskeletal conditions such as fractures, infections, and tumors.

## 5 Italy

On March 11, 2020, the World Health Organization declared a pandemic of a new type of coronavirus disease, COVID-19, which is responsible for symptoms that included a severe acute respiratory syndrome.^[30]^ Italy was one of the first countries in the world to have active COVID-19 cases. The first case was diagnosed in Lombardy (Codogno, February 20, 2020),^[31]^ and the disease spread uncontrollably throughout the entire county, accounting for 239,000 cases and 34,767 deaths by June 30, 2020. The region Emilia-Romagna, became the third Italian region with a major number of cases (after Lombardy and Piedmont) with 28,492 positive patients and 4260 deaths by the end of June, 2020.^[32]^

On January 31, the Italian Council of Ministers declared a national state of emergency related to health risks for 6 months. On February 21, the Minister of Health quarantined those who had been in contact with COVID-19-positive people and activated surveillance and home stays for those who had traveled to the risk areas in the previous 14 days. To contain the infection, the areas with the highest number of cases were isolated. The so called “Lockdown” was declared by the Government on March 9 throughout the Italian territory and the Italian Phase 1 began.

### 5.1 Organizational actions made to the trauma services

Our Regional Health Care Systems, such as Emilia Romagna, and the entire Italian National Health Care System, had to quickly adapt its organization to meet the needs of COVID-19-positive patients. It was decided to establish a hub-and-spoke organizational design, identifying regional macro area centers of excellence in infectious diseases. Specifically, hubs had the capacity for extracorporeal membrane oxygenation; spokes, instead, were secondary treatment institutions that offered more limited services. Hubs, with active 24-hour-a-day services, had different pathways for COVID-19-positive and negative patients, and several specific hubs were selected as COVID-19 free for trauma, neurosurgical, neurological, and cardio-vascular emergencies.

### 5.2 The Emilia-Romagna network

In April 2020, the Emilia-Romagna network became a national referral network for intensive care unit (ICU) management, establishing protocols for ICU COVID-19 therapy. The network had 146 beds that could be used to assist infected and symptomatic patients. Within the Emilia-Romagna network, the Ravenna Hospital—Area Vasta Romagna was identified as a hub center and promptly reorganized. Surgical activity was rationed, cancelling all elective surgery and performing only trauma cases to minimize the potential overlap of COVID-19 patients with postoperative COVID-19-negative patients.

During Phase 1, the ICU was converted in a COVID-19 ICU area, while some of the operating rooms, normally used for elective surgery, became a COVID-19-free ICU. A collaboration network was created between the ICUs of the entire Region to maximize the care of those patients who needed mechanical ventilation. The anesthesia groups and emergency room staff were increased to serve the network. Novel care pathways were created, starting at the triage area. Every patient was given a COVID-19 questionnaire to search for possible flu-like symptoms or contacts with infected individuals during the previous 14 days. Vital signs were taken. Patients with negative medical histories, no respiratory symptoms, temperatures </=37.4°C, and no COVID-19 contacts were placed in a clean “green area,” wearing only standard PPE consisting of surgical masks and gloves. Patients with suspected symptoms or recent contacts with COVID-19-positive individuals were placed in a “gray area, where FFP2, protective gowns, goggles, and gloves were used. For those patients who needed hospitalization, based on the outcome of the chest CT scan and oropharyngeal swab, specific pathways with distinct corridors and wards were created; clean green rooms for the COVID-19-negative patients, red rooms for the COVID-19 patients, single-gray rooms for the isolation of surgical/nonsurgical patients and those waiting for an oropharyngeal swab report.

With regards to orthopaedic emergencies, Phase 1 brought a reduction of 76% in weekly emergency room access compared with the same period in 2019; 57 orthopaedic traumas per week presented in March to April 2020, compared with 234 traumas per week in March to April 2019. A substantial change in traumatic mechanism-of-injuries was noticed, with a considerable increase in indoor accidents relative to outdoor ones (e.g., traffic-related, sports-related, and occupational accidents). Out of the 8 operating rooms in our Department, only 3 were reserved for the “COVID-19-free surgery” and one was set up for COVID-19 patients, with medical personnel trained to manage these patients to reduce the risk of aerosols and droplets related to intubation for orthopaedic operations. Where possible, local or spinal anesthesia was used. The remaining 4 rooms were converted to COVID-free ICUs. Fracture patients in need of surgical treatment were moved to the COVID-19-free or COVID-19 OR depending on their swab result. Patients who presented to the emergency room with a severe acute traumatic condition and required immediate surgical treatment were moved directly to a COVID-19 OR, being considered potentially infected. Entire wards for COVID-19 patients with simple vital sign monitoring capacity were created, bringing together patients from all surgical departments in a single sector.

With a lack of internists and pneumonologists to provide urgent 24-hour care, surgical specialists were recruited to assist with the care of these patients under the guidance of an internist. Orthopaedic surgeons also were recruited for this service, creating the “ortho-pneumologist.” Visiting hours access was not permitted for anyone, including for those COVID-9-negative patients. On March 11, as with all elective surgery, all orthopaedic nonurgent outpatient visits were suspended. Only patients who were postdischarge and those who needed cast removal were seen after having completed the COVID-19 questionnaire screening. From May 4 to June 3, a reduction was seen in the rates of infection and the government instituted the so-called Phase II, characterized by the opening of businesses inside the regional borders. During this new phase, the hospital organizations maintained their Phase I pathways.

As of the writing of this article in late June, we are currently in the so-called Phase III. Phase III is characterized by the gradual restoration of both elective surgery and outpatient clinics, although with some restrictions. Elective orthopaedic visits resumed according to the expertise established by each institution. The daily number of visits was halved to avoid gatherings and allow the disinfection of the rooms. Elective orthopaedic surgical care was reorganized. Each patient had to undergo the COVID-19 screening questionnaire, an oropharyngeal swab test, and a chest x-ray the day before hospitalization; only COVID-19-negative patients were allowed to have surgery. All fracture patients who needed a surgical procedure, even those without symptoms and with a negative COVID-19 history, were required to have a chest x-ray and COVID-19 swab test in the emergency room for screening purposes. The patient was kept in functional isolation until the test results returned. However, for patients who needed urgent interventions, a 2-hour rapid test was performed or the patients were treated according to the COVID-19 protocol.

### 5.3 Other important/unique experiences and lessons learned

This COVID-19 pandemic has transformed the medical practice in Italy in many ways. Since its inception, the community of Italian physicians noted significant decreases in cancer diagnoses and biopsies by 52%, delays in surgery by 64%, decreases in patient visits per week by 57%. At the moment, the physician community is unable to predict the progress of the pandemic during Phase III and will adapt their practices relative to the curves of the infection rates.

In anticipation of a possible spike in cases in Italy in the upcoming winter, 2 maxi ICU, located at Maggiore Hospital (Bologna) and Infermi Hospital (Rimini), are being created; they will function as hub centers for the intensive care of COVID-19-positive patients for the entire Emilia Romagna Regional System.

## 6 Greece

The management of the COVID-19 pandemic constitutes a significant and multifactorial challenge for every country's health care system. To date, Greece is considered to have successfully managed the COVID-19 pandemic. As a result, the detrimental effects of the pandemic on public health and the health care system have been successfully mitigated. Since the outbreak of the pandemic through June, 2020, 3589 cases of COVID-19 have been reported in Greece, with 193 deaths and 121 patients successfully discharged from intensive care units after being admitted due to COVID-19.^[33]^ The majority of the cases (54.7%) involved male patients.^[33]^ The mean age of the infected patients is 47 years, and the mean age of the deceased patients is 76 years.^[33]^ In 95.9% of the deceased patients, there was at least 1 comorbidity present and/or the patients were older than 70 years.^[33]^ The increasing number of COVID-19 cases and the formation of a steep infection curve lead the Greek government to enforce lockdown measures on March 14, 2020, following the recommendations of the infectious diseases specialists and the National Public Health Organization. The lockdown period lasted until May 4, 2020. From that date onward, the lockdown measures were gradually started to be withdrawn.

During the lockdown period, transportation restrictions and social distancing were implemented. Additionally, Greek citizens were strongly advised not to leave their home unless it was absolutely necessary, including for health care visits. The patients were encouraged to communicate with their physicians by telephone and proceed to visit them for a face-to-face consultation only after clear instruction by their family physician. Additionally, the online drug prescription software was modified to allow the prescription to be received as an email and not in paper as was the usual standard before the pandemic, with the goal of keeping health care visits to a minimum. In addition, the outpatient departments of the hospitals were shut down and Greek citizens were advised to avoid visiting the hospital emergency departments unless it was a true emergency. Strict measures were enforced at the hospitals’ entrances, where patients who presented with conditions that did not require emergency care were not allowed to enter the hospital. Strict measures were also applied to visitors of admitted patients; only 1 visitor was allowed per day and only for a limited period of time.

### 6.1 Organizational actions made to the trauma services

In relation to trauma cases, special operating rooms were assigned for the management of COVID-19 patients. Routine COVID-19 testing was not performed in trauma and fracture patients. If clinical symptoms related to possible COVID-19 infection were evident in a patient, the treating orthopaedic physicians communicated with the dedicated COVID-19 infection control group of their hospital, which decided whether testing was necessary. As mentioned above, COVID-19 patients were treated in dedicated operating rooms with all necessary precautions according to the national guidelines.^[34]^ During the first month of the pandemic in Greece, there was shortage of PPE, not only for civilians but for hospital personnel as well. The official personal protection equipment policy for health care workers included the requirement to wear surgical masks at all times in their workplace. In the majority of departments, each worker was handed 1 surgical mask per day. National guidelines/instructions regarding other items of PPE were applied only for the personnel of dedicated COVID-19 units or those involved in aerosol-generating procedures such as endotracheal intubation.

The management of the pandemic required the introduction/allocation of new units dedicated to COVID-19 management within the existing health care facilities, that is, the hospitals. Despite hiring new hospital staff dedicated to pandemic management, it was not possible to substantially increase the number of health care workers that were needed to manage the pandemic. As a result, repurposing of the existing personnel was necessary. In trauma and orthopaedic departments, the transfer of health care personnel only included a section of the nursing staff. Medical personnel were not repurposed, as the pandemic in Greece did not reach the extreme extent that would require all available medical personnel to be transferred to the management of COVID-19 patients, similar to what happened in the neighboring country of Italy. The repurposing of medical personnel included only internal medicine physicians and was not extended to surgeons.

### 6.2 Effect on clinical care delivery from trauma service

Road traffic accidents are the main cause of major trauma in Greece. During the lockdown period, which involved the second fortnight of March and all of April, the number of accidents was drastically decreased; therefore, the number of trauma cases was decreased as well. For example, in the University Hospital of Heraklion, Crete, which is a tertiary referral center in a region of 634,000 citizens during winter and over 2.5 million people during summer, only 1 case of major trauma requiring orthopaedic surgical management was treated during the lockdown time.

The triage of trauma cases was not affected by the pandemic. Patients who required emergency care underwent an operation as soon as possible without the necessity for COVID-19 testing. The same standards were applied for fractures that required urgent care, such as open fractures or fractures of the pelvis and femur. Fractures that did not require urgent care and could be treated in a staged manner were managed according to the local hospital policies. Dedicated trauma operating rooms do not exist in every hospital in Greece. In hospitals with dedicated trauma rooms, trauma cases were assigned to each trauma list according to the schedule. In a hospital without dedicated trauma rooms, fractures were treated as soon as a room was available. As mentioned above, there was not routine preoperative COVID-19 testing for trauma and fracture patients. All patients who underwent an operation during the pandemic were transported to the operation theater wearing surgical masks. If the patient underwent general anesthesia, the mask was kept on while bag mask ventilation was performed and then it was removed for the endotracheal intubation to be performed. In cases where the patient underwent regional anesthesia, a surgical mask was used during the whole procedure. In cases where surgery was performed in a verified COVID-19-positive patient, the anesthesia team was advised to use personal protection equipment that included: high level of protection mask (FFP2/FFP3/Ν95/ΚΝ95), eye and face protection equipment, long sleeved waterproof gowns, and disposable gloves. The surgical team was equipped according to the usual standards.

The outcome of trauma cases was not significantly affected by the COVID-19 pandemic, as the approach of the health care system to trauma cases was not modified. However, it should be noted that the decreased number of trauma cases offered the possibility for earlier treatment of fractures where otherwise time to surgery would be significantly longer due to unavailability of operating room time. Therefore, patients with hip and other fragility fractures underwent an operation sooner and, as a result, their duration of hospital stay was significantly decreased.

### 6.3 Other important/unique experiences and lessons learned

Overall, the COVID-19 pandemic and especially the lockdown period in Greece resulted in a decreased workload of trauma units, as the strict measures of citizen transport control resulted in decrease of motor vehicle collision events. The number of fragility fractures was also decreased due to social distancing measures that particularly affected elderly people. The outcome of trauma patients was not affected as the management approach of aforementioned patients was not altered. The fact that postlockdown there was a significant increase of polytrauma patients due to motor vehicle collision events highlights the significant morbidity and mortality of road traffic injuries in Greece. Nevertheless, the decreased number of trauma cases in combination with the interruption of elective surgical procedures had a negative impact on residents’ education and their exposure to the number of major trauma cases, which could have been greater if the duration of the lockdown measures had been extended.

## 7 Switzerland

In Switzerland, the first cases of COVID-19 were detected in early February 2020. The number of cases was influenced by the Italian experience in mid-February and March, particularly in the most southern area of Tessin on the Italian border. The border to Italy was rapidly closed. Nevertheless, the Tessin area continued to be the most affected area throughout the first wave. The other region with the highest case load was on the French border. As of July 7, 2020, 32,369 persons had tested positive and 1686 persons had died due to the virus or through virus-related complications.

In the hospital, the lockdown included a strict limitation of visitors, which was extended through at least July, 2020. Certain hospital entrances were closed, with only the main entrances used for outpatients with appointment letters. The ICU capacity for COVID-19 was doubled by reorganizing personnel from other Departments that had had previous ICU experience. One ICU was strictly reserved for COVID-19 patients and a shelter was opened. In-person teaching and training activities were stopped.

### 7.1 Organizational actions made to the trauma services

In early March, Trauma Departments were split up in groups of physicians that performed weekly services, limiting contact between services. Social distancing was also adopted, with larger rooms for patient care conferences. Masks were mandatory for all personnel and patients, including during transport within the hospital.

The number of trauma cases dropped by more than 50% until late April/early May, 2020. During this time, the federal health department recommended that individuals stay at home and the government closed nonessential businesses (e.g., clothing stores, bars, and restaurants), which had an impact on daily practice. During the pandemic, approximately one-third fewer patients were treated in the trauma bay. Despite a comparable number of polytrauma patients, the average ISS was significantly lower during the pandemic.

Following the lockdown and subsequent interruption of planned surgeries, the Swiss Department of Health decided to reopen the operating rooms for elective surgeries on April 27, 2020. In preparation for these measures, multiple preparations were undertaken. Among these has been a written statement to inform patients and health care workers about preventive options. These statements appeared to be required for the population as there were reports of patients expressing concerns about possible hospital-acquired COVID-19 infections. Video-based outpatient clinics were offered and some elective surgeries were cancelled indefinitely or nonoperative options were pursued.

Within the health care staff, precautions were maintained throughout the lockdown and thereafter. These included: mandatory mask-wearing at all times on hospital campuses for health care workers; mandatory masks for patients while being transported inside the hospital; no visits by relatives, unless a life-threatening condition has occurred (this measure was gradually loosened up as of July 1); limitation of the number of persons in changing rooms, restaurants (that had been closed during lockdown); use of a testing suite for personnel and patients at risk; hospital access limited to only those patients who had verifiable appointments; and hospital access limited to certain entry areas, with separate entrances and exits.

### 7.2 Other important/unique experiences and lessons learned

The combination of strict lockdown rules, strict rules within hospitals, and distinct reopening rules were all recognized as being helpful in keeping the case load tolerable and the Swiss health care system afloat. Of note, wearing a mask in public was not reinforced until early July, 2020 in Switzerland, when certain areas developed secondary regional spikes. Continuous awareness will be crucial in the future to avoid further spread.

## 8 Germany

Germany observed the first cases of COVD-19 toward the end of January, 2020. Initially, there were 3 hotspots: one at a company in the south that had a close relationship to an exchange program with China, 1 after a large event party in the west, and 1 from returning ski tourists from an Austrian ski resort. Since that time, the virus spread over Germany and affected all provinces. In the first weeks, hospitals took individual measures to prepare for possible admissions of infected patients. On March 11, 2020 the World Health Organization Director-General declared COVID-19 a pandemic. On March 12, 2020 the German Minister of Health requested that all hospitals postpone elective admissions and surgeries. The aim of this request was to increase capacities for possible hospital admissions and ICU treatment of the expected COVID-19 patients. A special “COVID-19 hospital support law” was approved by the government that regulated financial compensations for hospitals.

### 8.1 Organizational actions made to the trauma services

As of March 16, elective admissions and surgeries were cancelled in most of German hospitals. Previously scheduled surgeries were postponed. Many discussions ensued regarding the definition of “elective surgery.” With respect to trauma and orthopaedics, most hospitals and surgeons accepted the definition that all emergency care and surgeries that would negatively affect the outcome of the patient if postponed for several weeks or months should be performed. Typically, all fractures, infections and malignancies were admitted and treated. All elective joint replacements, most arthroscopic interventions, including ACL reconstructions or staged rotator cuff repairs, and most spine surgeries were postponed. This was generally well accepted by patients and their families as there was a fear of acquiring COVID-19 in the hospital. Depending on the type of department, there was a surgical case reduction of 60% to 80% due to the decreases in both trauma and elective cases. Most hospitals observed a reduction in emergency room visits up to 50%, most due to minor medical conditions, as patients feared COVID-19 contamination. The number of emergencies with relevant trauma, such as fractures, remained the same.

The main concern of politicians and hospital administrators was that the resources for treatment of COVID-19 patients would not be sufficient if the virus spread exponentially. Therefore, they proceeded with a 2-target strategy. The first target was to reduce possible contact with potentially infected people. This led to the general lockdown in Germany on March 16, 2020. In German hospitals, the directive was to reduce all potential contacts between people. All nonemergent outpatient contacts were stopped immediately. Inpatient visitors were not permitted, with the exception of those family members that needed to visit their children or terminally ill relatives. All medical personnel, patients, and visitors were required to wear masks. Further, minimum distances of 1.50 m had to be maintained, including in waiting areas and the recovery room. Daily conferences and rounds were reorganized to reduce the number of participating residents and staff. Some hospitals reorganized their meetings using web-conferences for their rounds and educational courses. In many hospitals, their teams were reorganized weekly to reduce potential cross-contamination of their teams. All patients who were transferred to rehabilitation centers needed a negative COVID-19 test within 72 hours before discharge.

The second target was to increase resources for the treatment of COVID-19 patients. Therefore, elective surgery and admissions were stopped to free potential ICU resources and provide the chance to re-educate health professionals to support ICU teams. Additional ICU resources were built up, including converting ORs into ICUs. As residents in trauma/orthopaedics routinely rotate through the ICU for 6 months during their residency, all those who had been to ICU in the past 3 years were reinstructed for a potential rotation to the ICU if necessary. A national ICU bed registry was established at the beginning of the crisis to keep track of which ICU resources were still available.

Though the number of available ICU beds was never an issue, there was a problem with not having sufficient masks, gowns, and COVID-19 tests. Most hospitals held daily meetings of their hospital administrations to address these problems and develop solutions. Prior to the crisis, Germany already had a generous number of ICU beds (34 beds per 100,000 inhabitants). This number was further increased during the pandemic. Because of the effective measures taken during the lockdown the available resources were never used completely. This made it possible to provide support to other countries and to treat patients transferred from Italy, France, and the Netherlands to German hospitals.

COVID-19 patients who presented with trauma had surgical procedures as necessary with all the required precautions taken by the treating staff. COVID-19 patients with trauma were treated on COVID-19 wards or ICUs. All staff that presented with fever and cough were tested. COVID-19-positive staff stayed home for at least 14 days and needed a negative COVID-19 test before being allowed to return to work. With these measures, very few hospitals observed COVID-19 outbreaks in patients or staff.

On April 27, 2020, the Minister of Health announced that hospitals should plan to begin to increase their elective admissions and surgeries. In mid-May, 2020, most hospitals steadily increased their number of surgeries, hospital admissions, and outpatient visits. Visitors were allowed to enter the hospital again, albeit 1 per patient per day for 30 to 60 minutes. All patients and staff continued to wear masks. In 50% of hospitals, all elective admissions and surgeries required patients have a negative COVID-19 test within the last 96 hours. Patients with emergent conditions that needed orotracheal intubation and did not have a negative COVID-19 test required special precautions (face shields and FFP2 masks) by the anesthesiologist. At the end of June, 2020, most hospitals were back to 90% of their prepandemic patient volumes.

As of July 5, 2020, Germany, with a population of 84 million, had 197,418 registered infections, of those 181,700 have recovered and 9081 died. The initial peak of COVID-19 infections in Germany were in mid-April 2020, which was followed by a steady decline. The number of tests performed was 70,000 per 1 million inhabitants.

### 8.2 Other important/unique experiences and lessons learned

The COVID-19 pandemic was a challenge for the German health system. Because of the dramatic developments in neighboring European countries, the German Minister of Health took significant action on March 12, 2020 and requested all hospitals to postpone nonemergent surgeries and hospital admissions. Hospitals followed these requests, increasing their efforts to reduce potential contact with COVID-19-infected patients, and freeing up available resources for the treatment of COVID-19 patients. Trauma and orthopaedic surgery services observed a 60% to 80% reduction in their patient volumes, reflecting the decrease in number of trauma cases and elective surgery. During the crisis, the number of ICU beds, respirators, and staff were never a problem. There was a problem, however, related to the shortage of available masks, gowns, and COVID-19 tests initially. The Minister of Health has agreed to financially compensate hospitals for cancelled elective surgical procedures and hospital admissions.

## 9 Netherlands

The first person in the Netherlands, a country with a population of17.5 million inhabitants, to test positive for COVID-19 did so on February 27, 2020. Hospital admissions for COVID-19 peaked exactly 1 month later on March 27 and ICU admissions a week later. By June 1, 2020, according to the National Institute for Public Health and the Environment (RIVM), 46,545 people had tested positive, 2869 cases required admission to an ICU, and 5962 died. When comparing the periods’ mortality rate with a baseline using the previous years, excess mortality was estimated at 8600 cases. Through April 30, 2020, almost 14,000 positive cases (35% of total) presented amongst health care workers, 9 of whom have passed away.

To guide government policy in situations transcending pre-existing protocols, a dedicated expert panel (OMT) became operational on January 24, 2020. At the beginning of February, 2020, the chances of the disease posing a significant threat to Dutch public health were thought to be small and the country was deemed to be well prepared. The first cases in the Netherlands were all traced back to recent visits to northern Italy. At the beginning of March, 10 people had tested positive. This number rose past 1000 on March 15 and past 10,000 on March 29. The burden of disease was most pronounced in the southern provinces, possibly due to the Netherland's phased system of national holidays and return of many people from trips to skiing-areas in the Alps with widespread infection. The month was characterized by a scramble for measures to contain the spread of the virus. By March 9, general hygienic measures were proposed by the government and people in affected areas were recommended to work from home where possible. The initial strategy in trying to contain the outbreak was through contact tracing and quarantining, partly instigated by insufficient testing capacity. By March 23, the level of measures reached its peak. A state of “intelligent lockdown” was announced that would continue until its phased relaxation starting May 11. Schools, nurseries, and the hospitality industry were closed down. People were called upon to self-quarantine when sick or potentially exposed and to practice social distancing. Shops and delivery services were allowed to remain open conditionally. Medical screening programs were paused. Hospital visitation was severely restricted and nursing home and other care facility visitations were put on hold.

### 9.1 Organizational actions made to the trauma services

Fortunately, the crisis never reached the point requiring shifts in indications for treatment or where changes in patient triage were necessary. Through our scientific societies, experiences in crisis management were shared and advisory guidelines were created. Webinars played an important role, instilling a generalized sense of urgency and sharing important lessons early on, when some hospitals, particularly in the south of our country, were becoming overwhelmed by the surge in COVID-19 cases, while others had yet to experience the pressure.

By March 15, the first recommendations of the Dutch Surgical Society, amongst others, included creating extra capacity to admit and care for patients by reducing elective care and repurposing existing infrastructure, critical appraisal of the use of PPE in light of pending shortages, and coordinating regionally for a collective approach. Further recommendations covered separating COVID-19 patient streams, standardizing COVID-19 treatments, centralizing coordination of human resources, optimizing IT support, and working toward national triage criteria. Regional coordinators were appointed per surgical subspecialty to facilitate the transfer of care from hospitals where COVID-19 care had supplanted surgical care. Guidelines were published that denoted which elective/semielective surgical care, ranging from trauma to oncology, could acceptably be postponed and to what degree.

Modeling of regional and national redistribution of trauma cases was made and, by March 21, the government started coordinating the national redistribution of both equipment and patients requiring critical care to better match capacity and demand. Over 200 ICU patients were transferred between hospitals, 60 of whom were transferred to a very helpful Germany. Between March 16 and 31, total ICU capacity was increased from 1150 to 1900 beds. By April 5, further preparations of personnel and equipment were in place to realize a subsequent step up to 2400 ICU beds, should demand have arisen. Ultimately, ICU demand peaked on April 7, with roughly 1400 COVID-19 and 250 non-COVID-19 patients.

Procurement of PPE and medical equipment was nationalized beginning on March 24. Standard type IIR surgical masks were considered sufficient to treat infected patients and FFP2 masks were only indicated for use during high-risk, aerosol-generating procedures. Procedures for sterilization and reuse of masks were developed.

In the early stages of lockdown, a downward trend in the number of trauma cases was noted, possibly due to reduced driving and participation in sports. Currently, data from this and last years are being reviewed to establish a more objective picture of changes in case-mix and outcomes.

Several factors affected the limits on surgical capacity. Chief amongst these were the redistribution of anesthesiological and OR personnel to manage the newly created ICU beds, ICU capacity for postoperative care in complex cases, and availability of beds on the regular wards. Reserves in medical equipment and PPE ran low, but did not run out. Implementing separate patient streams with dedicated teams through the entire chain of care stressed both human resources and available space.

To mimimize in-hospital transmission and to ensure a safe work environment, national guidelines for preoperative screening methodologies were frequently updated according to testing capacity and clinical evidence. Initially, PCR and low-dose chest-CT for every asymptomatic patient was recommended. Patients were treated as infected if the test results were unavailable at the time of surgery. After the initial results of the SCOUT-1 trial pointed to the relatively minor contribution of CT scan versus PCR in diagnosing COVID-19, standard scanning was abandoned. Increased availability of fast PCR testing with turnaround times of less than 2 hours has made preoperative testing easier recently.

Diagnostic procedures and therapeutic interventions were planned to minimize hospital stay and limit in-hospital movement. Nonessential clinical activities were avoided. Whenever possible, outpatient clinics were handled by phone and, when nonoperative fracture management was initiated in the emergency department, immediate definitive casting was performed by specialists aiming to decrease the amount of intermediate return visits.

The national testing capacity was limited early in the course of the epidemic and was initially reserved for high-risk groups. The scope of eligibility was set by the RIVM and widened with growing availability, which grew slower than in most surrounding countries, until everyone could be tested by June 1. In reality, testing never reached the laboratories’ capacity at any point.

### 9.2 Other important/unique experiences and lessons learned

Initially, the ferocity with which COVID-19 would hit our society was seriously underestimated by the majority of health care workers and policymakers alike. Its incubation time gave the virus a 2-week head start on policy and its exponential growth meant that even a small delay in measures would have an explosive effect down the line. Fortunately, within a month of the first established case, COVID-19 incidence had peaked and the curve was flattened, without forcing physicians to lower our standards of emergency care. Our medical professionals are used to working in a transparent health care system with national quality registries and intensive regional and national collaborations. Experiences from abroad hit close to home and illustrated the importance of grave measures while our national epidemic was still in its early stages. Webinars helped disseminate experiences and ideas effectively. Together these allowed for a swift implementation of wide-ranging measures, preventing worse outcomes. Future research will demonstrate which of those measures were most meritorious. Hopefully, in the future, intelligent approaches to health care during a pandemic will triumph over sheer restriction.

## 10 Conclusions

These reports highlight the immense impact of the pandemic on orthopaedic and trauma services all over Europe. The rapid evolution of practice and knowledge allowed services to adapt and respond to the challenges. Concerns for personal safety, stopping the spread of the virus, and the delivery of necessary urgent care for patients led to new practices and understanding. The reports highlight how elective surgery was universally stopped, surgical services were reconfigured, and new practices, such as the widespread use of telemedicine, may well become permanent. It also emphasizes how the pandemic has re-educated us on the importance of a consistent and central approach to deal with a global health crisis, and how medical services need to remain flexible and responsive to new ways of working.
